# Effects of onabotulinum toxin type A injections in patients with Meige's syndrome

**DOI:** 10.1055/s-0044-1785691

**Published:** 2024-04-19

**Authors:** Alexia Duarte, Léo Coutinho, Francisco Manoel Branco Germiniani, Hélio Afonso Ghizoni Teive

**Affiliations:** 1 Universidade Federal do Paraná, Setor de Ciências da Saúde, Curitiba PR, Brazil.; 2 Universidade Federal do Paraná, Programa de Pós-Graduação em Medicina Interna, Curitiba PR, Brazil.; 3 Universidade Federal do Paraná, Hospital de Clínicas, Departamento de Clínica Médica, Setor de Neurologia, Curitiba PR, Brazil.

**Keywords:** Meige Syndrome, Dystonia, Movement Disorders, Botulinum Toxins, Síndrome de Meige, Distonia, Transtornos dos Movimentos, Toxinas Botulínicas

## Abstract

**Background** Meige's syndrome is a type of facial dystonia characterized by the simultaneous occurrence of blepharospasm and oromandibular dystonia. Although botulinum toxin type A (OBTA) injections are the standard treatment, evidence of their effectiveness and safety in this scenario is still lacking.

**Objective** Our research aimed to evaluate the improvement and occurrence of side effects following injections of onabotulinum toxin type A (OBTA) in patients with Meige's syndrome.

**Methods** Patients with Meige's syndrome undergoing botulinum toxin injections were enrolled in this study. We assessed dystonia intensity before and 14 days after OBTA injection using the Burke-Fahn-Marsden Dystonia Rating Scale (BFMDRS) to measure the response of symptoms in the eyes (blepharospasm) and mouth (oromandibular dystonia). Other variables, such as dosage, side effects, and demographic data, were also recorded.

**Results** The study included 41 participants, with a mean age of 67.7 years and a female-to-male ratio of 3.5:1. The mean BFMDRS score before the injections was 8.89, and after 14 days, it was 2.88. The most reported side effect was ptosis, with a 7.3% incidence. OBTA significantly reduced dystonia severity ( *p* < 0.0001). The clinical response for the blepharospasm component was superior to the oromandibular dystonia component.

**Conclusion** Our results support that OBTA seems to be an effective and safe therapeutic option for treating Meige's syndrome. The effect of OBTA was more pronounced in the treatment of blepharospasm than in oromandibular dystonia.

## Resumo

**Antecedentes** A síndrome de Meige (SM) é caracterizada pela ocorrência concomitante de blefarospasmo e distonia oromandibular. Embora a toxina onabotulínica do tipo A (TBA) seja o tratamento de escolha, há uma falta de evidências sobre sua eficácia e segurança nesse cenário.

**Objetivo** O objetivo do nosso estudo foi avaliar os efeitos obtidos com a aplicação de TBA em pacientes com SM.

**Métodos** Pacientes com SM que realizam aplicação de TBA foram convidados a participar desse estudo. Os participantes foram questionados sobre a intensidade da distonia antes e 14 dias após a injeção de TBA, utilizando a Escala de Distonia de Burke-Fahn-Marsden (EDBFM) para mensurar a resposta obtida em cada segmento. Outras variáveis, como dose, ocorrência de efeitos colaterais e dados demográficos, também foram registradas.

**Resultados** O estudo contou com 41 participantes (idade média de 67,7; razão de 3,5 pacientes do sexo feminino para cada participante do sexo masculino). O escore médio na EDBFM antes das aplicações de TBA era 8,89, e, após 14 dias, 2,88. O efeito colateral mais reportado foi ptose (7.3%). A TBA foi capaz de reduzir a severidade da distonia ( *p* < 0.0001), principalmente do blefarospasmo.

**Conclusão** Nossos resultados corroboram que a TBA é uma terapêutica eficaz e segura no tratamento da SM. O efeito da TBA é superior no manejo do blefarospasmo em relação à distonia oromandibular.

## INTRODUCTION

Dystonia is a movement disorder characterized by involuntary muscle contractions, causing intermittent movements, the maintenance of abnormal postures, or both. 1 Within the spectrum of cranial dystonias, Meige's syndrome presents as the simultaneous occurrence of oromandibular dystonia and blepharospasm. Other movement disorders, such as cervical dystonia, might be associated with this condition. 2 This clinical presentation's first report in literature was in an article from the American doctor Horatio Wood, in 1887, but it was only in 1910 that the French neurologist Henri Meige published a detailed case series describing the simultaneous occurrence of blepharospasm and oromandibular dystonia. In the following years, different researchers would refer to the disorder as either “Wood's syndrome” or “Meige's syndrome,” honoring the two doctors. In 1976 the British neurologist David Marsden identified signs of facial dystonia in the painting De Gaper, by the Renaissance artist Pieter Brueghel. Due to his findings, the syndrome received a new name: “Brueghel's syndrome.” While some maintain they are the same, others argue that there are some differences in their clinical presentation. 3

Although the first description of Meige's syndrome dates back to the 19 ^th^ century, its pathophysiology remains unclear. The most accepted hypothesis suggests that it's the result of a hyperactivation of the striatum and hypothalamus, due to abnormalities of the dopaminergic system. 4 Conversely, new studies propose that there is an underlying impairment of brain networks, especially the basal ganglia-thalamocortical motor circuit 5 and right (pre)motor- and left occipital-thalamic streamline connectivity. 6

Genetic (the genes *THAP1* and *GNAL,* for example) and environmental factors have been linked with Meige's syndrome development. 2 4 However, the diagnosis remains mainly clinical. 7 Currently there is no cure for Meige's syndrome and available treatments include deep brain stimulation, oral medications, and botulinum toxin injections. 2 Oral medications (comprising anticholinergics, levodopa, neuroleptics, GABA receptors agonists, and atypical antipsychotics) may have a modest response in dystonia treatment, with a high incidence of side effects, therefore being poorly tolerated in the long-term. 8 Deep brain stimulation is a treatment option usually restricted to refractory cases, as it is an invasive procedure. 9

Botulinum toxin injections act in the presynaptic terminal of the neuromuscular junction of striated muscles through cleavage and inactivation of SNARE (Soluble *N* -ethylmaleimide-Sensitive Factor Attachment Proteins Receptor) protein complex, which is vital to the fusion of vesicles containing neurotransmitters to the plasma membrane in the synaptic cleft. Without the SNARE proteins, the fusion of vesicles and release of acetylcholine into the synaptic cleft do not occur, resulting in blockage of the neuromuscular junction. 10 For the treatment of blepharospasm, the target muscles are orbicularis oculi, corrugator, procerus, and frontalis, while for oromandibular dystonia, the chosen muscles are the masseter, temporalis, pterygoid, and digastric. The most common side effect is excessive muscle weakness, leading to ptosis, lagophthalmos, and dysphagia. Severe complications are unlikely and include generalized weakness, allergic reactions, and flu-like symptoms. 11

Although botulinum toxin is currently the first-line treatment, 4 this status is mainly derived from scientific evidence from studies with other forms of cranial dystonias, such as hemifacial spasm and blepharospasm, and a lack of suitable treatment alternatives, as oral medications have poor tolerability and surgical treatments are invasive. There are few studies quantifying the effects - both collateral and therapeutic - of botulinum toxin application in patients with Meige's syndrome. 2 This study aimed to evaluate the effects and safety profile of onabotulinum toxin type A (OBTA) injections in patients with Meige's syndrome.

## METHODS

**Figure 1:**
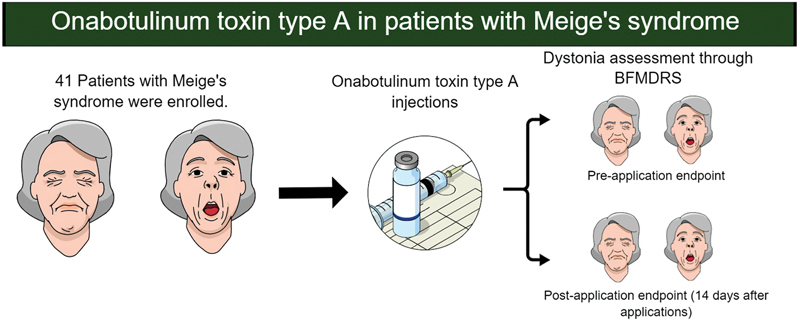
Infographic summarizing the flowchart for this study.

The present study was an open-label clinical trial. The enrollment and evaluation strategy for the study are summarized in Figure 1 .

### Eligibility and exclusion criteria

The inclusion criteria were patients over 18 years old diagnosed with Meige's syndrome by a neurologist specialized in movement disorders who undergo botulinum toxin type A injections every three months at the outpatient Movement Disorders Clinic of the Neurology Service at the Clinics Hospital of the Federal University of Paraná (Hospital de Clínicas da Universidade Federal do Paraná). We invited all patients who attended their consults in the period between March 16 ^th^ , 2020, and May 30 ^th^ , 2021, to compose the study sample.

The exclusion criteria were patients who refused to participate, did not understand, or did not sign the consent form, as well as those patients who were receiving other dystonia therapies.

### Assessment

We interviewed and examined the participants, applying the Burke-Fahn-Marsden Dystonia Rating Scale (BFMDRS) to rank dystonia intensity, 12 as the BFMDRS divides the body into segments affected by dystonia. In this study, we focused on the sections that pertained to Meige's syndrome, namely the “Eyes” and “Mouth” sections. Although a subset of our patients also presented with concomitant cervical dystonia, we did not include data on this segment as it was beyond the scope of this research. The BFMDRS scale graduates the “Provoking Factor” and the “Severity Factor” (both ranging from 0 to 4) for each segment. We multiplied the scores obtained from each factor and then multiplied the results again by a factor of 0.5, thus reaching the final segment score. Segment scores were then summed to reach the Total score:

([Eye provoking factor x Eye severity factor × 0.5] + [Mouth provoking factor x Mouth severity factor × 0.5] = Total score).

We questioned each participant about symptoms' severity and provoking factors before applying OBTA - considering 3 months after the last application as a Pre-Application endpoint (due to the washout period) - and afterward - considering 14 days after the application as the Post-Application endpoint. The same researcher (AD) conducted the interviews and converted the findings to BFMDRS scores. We also inquired the subjects about the occurrence of side effects and registered demographical data, such as the participant's age and sex. For some patients, data from more than one OBTA application were collected. In those cases, only side effects occurrence was noted.

### Botulinum toxin injections

The botulinum toxin used in this study was onabotulinum toxin type A (Botox, Allergan, Irvine, USA), presented in flasks containing 100 Units (U), diluted in 2 mL of saline solution. Injections were performed with 8 mm × 0.3 mm (30G) BD Ultrafine insulin syringes.

**Figure 2:**
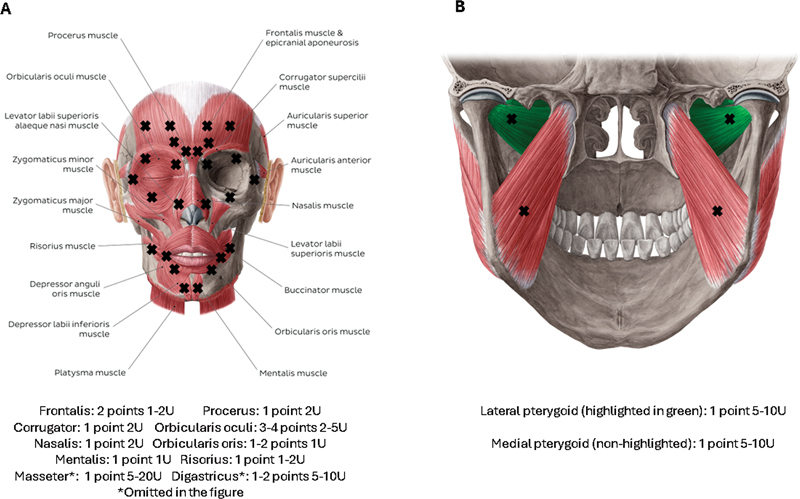
Injection sites and dosage. **A** : facial muscles; image used under license from Kenhub GmBH; illustrator: Yousun Koh; edited by the authors. **B** : pterygoid muscles; image used under license from Kenhub GmBH; illustrator: Yousun Koh; edited by the authors.

The same specialist assessed each patient about their symptoms, determined the injection points, and performed the applications. We individualized the selected muscles, tailoring them according to clinical presentation. We selected the target muscles as follows:

- Blepharospasm:
  ∘ for patients with eye closure: orbicularis oculi, corrugator, AND/OR procerus;
  ∘ for patients with intermittent blinking: same as above AND/OR frontalis;
- Oral dystonia:
  ∘ for patients with jaw closure: masseter AND/OR temporalis AND/OR medial pterygoid;
  ∘ for patients with jaw opening: lateral pterygoid AND/OR anterior belly of digastric;
  ∘ for patients with jaw deviation: lateral pterygoid AND/OR temporalis;

The basic injection protocol, with the toxin injection sites and dosage, is described in Figure 2 .

### Statistical analysis

Statistical analysis was performed with the software Jamovi 1.6 (The Jamovi Project, 2021) 13 14 and R 4.2.0. We used the Shapiro-Wilk test to determine the normality of distribution for numeric variables. Variables with normal distribution were described in terms of mean ± standard deviation. Variables with non-normal distribution were represented through median and interquartile ranges. Categorical variables are expressed in proportions and percentages.

A comparison of the two endpoints was established through a paired *t* -test for the variables distributed within normality and through a Wilcoxon signed-rank test for non-normal distribution. Statistical significance was defined as *p* < 0.05. We then measured the effect size through a corrected version of Cohen's d (Unbiased Cohen's d, also named Hedge's g), and 95% confidence intervals. We established the Hedge's g reference values to determine the effect size as 0.2 = small effect, 0.5 = medium effect, and 0.8 = large effect.

## RESULTS

We invited 54 patients to take part in this study, of which 13 patients met the exclusion criteria (7 did not accept or understand the study consent form, and 6 used other therapies for dystonia), leading to a final sample of 41 patients. Participants' ages ranged from 38 to 86 years old (mean age of 67.7 ± 12 years), with a marked predominance of females (9 men: 32 women).

**Figure 3:**
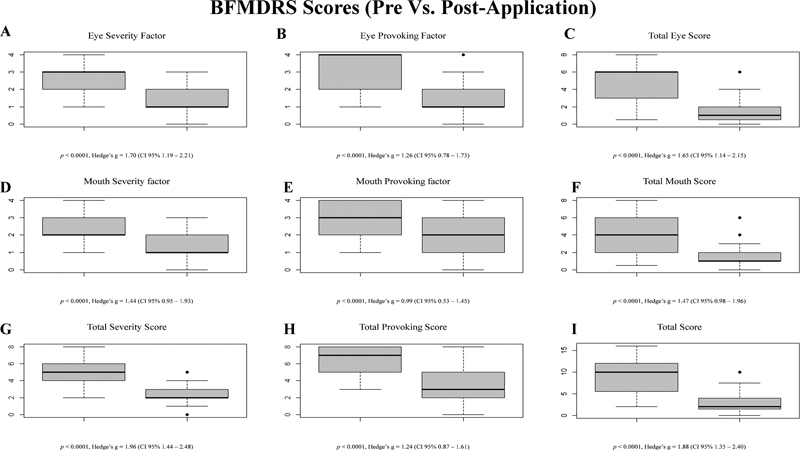
Boxplot graphics illustrating clinical response of dystonia as measured by the BFMDRS. Panels A-C correspond to the eye scores, panels D-F correspond to the mouth scores, and panels G-I correspond to the total scores.

For the eye scores, dystonia intensity as measured by the BFMDRS reduced from 4.7 ± 2.45 to 1.32 ± 1.46 (the severity factor reduced from 2.73 ± 1 to 1.17 ± 0.8, and the provoking factor reduced from 3.19 ± 1 to 1.7 ± 1.3). For the mouth scores, dystonia intensity reduced from 4.18 ± 2.23 to 1.54 ± 1.13 (the severity factor reduced from 2.48 ± 0.92 to 1.34 ± 0.61, and the provoking factor reduced from 3.17 ± 0.91 to 2.14 ± 1.10). The total score reduced from 8.89 ± 3.84 to 2.87 ± 2.29. All segments evaluated presented a statistically significant response with a large effect size, but we observed that the clinical response of the “Eye” segment was superior to the response in the “Mouth” segment (Hedge's g of 1.65 versus 1.47, respectively). Clinical responses with respective *p* values and Hedge'sg values are illustrated through boxplot graphics, in Figure 3 .

The mean OBTA dose applied was 58.38 U ± 22.17. Side effects occurred in 9.75% of the sample ( *n* = 4), the most common being bilateral ptosis ( *n* = 2), followed by unilateral ptosis ( *n* = 1) and facial asymmetry ( *n* = 1). All the participants considered the side effects mild and reported complete resolution within two weeks.

## DISCUSSION

Although demographic characteristics of our sample reflected the higher prevalence of primary dystonia among females (in accordance with the existing literature), the ratio obtained in this study (3.5 female: 1 male) is higher than those previously reported, which ranged from 1.43:1 to 3:1. 2 15 16 The age distribution observed in our study is also consistent with the literature, as Meige's syndrome is more common after the 6 ^th^ decade of life, 2 17 18 despite some reports of earlier onset, starting at the fourth decade. 19

The most common side effect found in this study was ptosis, with an incidence of 7.3% of the sample (considering both bilateral and unilateral ptosis). According to pre-existing data, ptosis is a common side effect of OBTA application, and its incidence fluctuates, with reported incidences ranging from 4% to 17%. 11 20 21 22 Facial asymmetry incidence in this study (2.4%) is also similar to that described by other authors. 21 However, lagophthalmos, dry eye, diplopia, eye-tearing, dysarthria, and dysphagia – also cited as frequent side effects 21 22 23 - were not mentioned by any participants of this study. In our service we routinely recommend that patients undergoing OBTA applications for Meige's syndrome and blepharospasm apply eye drops regularly, a fact that may have contributed to the absence of ophthalmologic complaints in our sample.

The side effects incidence ranges in the literature from 3% to 31.5%, 19 24 25 and the values described in our study (9.75%) are consistent with these reports. Strategies such as applications guided by ultrasonography and electromyography seem capable of reducing the occurrence of side effects 26 and optimizing therapeutic response, 27 but none of these methods were available in our study. Although adverse effects were frequent, they were self-limited and considered mild by the participants, reinforcing the notion that OBTA is a safe therapy for Meige's syndrome.

The interval between applications was 3 months. So far, this is the most widely used and accepted interval. However, many researchers in the field advocate shorter intervals. 24 The therapeutic effect following OBTA injection is perceived in 2 to 7 days, and it lasts for ∼3 months, 7 and this is the reason why this is the interval routinely used in our service.

The reduction in BFMDRS scores after OBTA application was higher in the eye section (g = 1.65, CI 95% 1.14–2.15) than in the mouth section (g = 1.47, CI 95% 0.98–1.96). Other studies also described this better responsiveness of blepharospasm when compared with oromandibular dystonia. 19 25 A possible explanation might be that the muscles involved in oromandibular dystonia are much more varied and situated deeper than the affected muscles responsible for blepharospasm. This fact renders the injections for oromandibular dystonia technically much more challenging than the injections for blepharospasm, as the muscles are more difficult to reach.

Despite the overall improvement in BFMDRS scores found in our study being comparable to prior reports of treatment with OBTA 19 and deep brain stimulation, 28 29 we should highlight several methodological particularities. The first aspect is our decision to evaluate only the facial component of dystonia. Although in our sample we included patients that presented comorbid cervical dystonia, in our service we use Abobotulinum toxin type A to perform injections in the affected muscles in cervical dystonias, which would by itself be a confusion bias. Therefore, we did not use the neck section of the BFMDRS to evaluate the participants or analyze the injection of botulinum toxin for cervical dystonia in this study.

In addition, the BFMDRS has some limitations as a tool in the assessment of dystonia if several segments are involved. The correction factor of 0.5 assigned to eye, mouth, and neck dystonia scores underestimates the disease severity in these segments when compared with trunk and limb dystonias. By using only the eye and mouth components, we eliminated this bias in our study and restricted our analysis within the scope of Meige's syndrome. The Unified Dystonia Rating Scale (UDRS) was recently presented as an alternative to solve this problem, 30 but we decided to use BFMDRS as it is more widely used in studies with cranial dystonias, as well as it is routinely used in our service for research and clinical purposes.

Another noteworthy aspect of our methodology is the use of Cohen's d /Hedge's g to determine the effect size. While other works preferred to express the degree of clinical response in terms of percentage, 19 we believe that presenting the effect size through these measurements might facilitate the compilation of evidence in future meta-analyses.

This study had several limitations. There is an information bias, due to the study design, which is based on patient answers. To minimize this bias, the same researcher did all the interviews and we used a standardized questionnaire. There was no control group to compare the results, but considering that botulinum toxin is currently the standard of care in segmental dystonias, a placebo-controlled study with OBTA could be considered unethical. In circumstances such as this, where to perform a double-blind study would deprive the patients of an established treatment for facial dystonias in general (although poorly studied in the context of Meige's Syndrome), with functional and social implications to the patients involved in these studies, open-label designs are a useful resource to better quantify the effectiveness and safety of a treatment, thus adding to the existing knowledge. Another shortcoming is the lack of standardization in OBTA dosage, as most patients were already on treatment, using individualized quantities. Finally, by evaluating only a 14-day post-application endpoint we did not include a significant variable: duration of effect. The inclusion of a 3-month endpoint could have provided this data. Further studies may be useful to establish the influence of dosage, duration of effect, and even laboratory evaluation of antibodies against botulinum toxin, as additional means to ascertain the outcomes of OBTA application in patients with dystonia. Our study did not take into consideration the specific phenomenology of the oromandibular dystonia components among our cohort of patients with Meige's Syndrome. As a result, we were unable to establish a subgroup analysis to characterize differences in the effectiveness of OBTA among the spectrum of phenomenological presentations (jaw opening dystonia, jaw closure dystonia, jaw deviation, tongue protrusion, among others).

In conclusion, our findings suggest that OBTA application is both an effective and safe therapy, capable of attenuating the symptoms of Meige's Syndrome. Side effects reported by our patients were mild, self-limited, and did not require any further treatments. Future studies are necessary to strengthen the evidence of OBTA use in Meige's syndrome.
